# Criteria-based outpatient scheduling at a nephrology clinic: prospective evaluation of patient pre-assessment and its corresponding adaptive scheduling strategy

**DOI:** 10.1186/s12913-024-11615-7

**Published:** 2024-09-28

**Authors:** Ruben Klaas, Jedidja Lok-Visser, Joan Doornebal, Ton Roelofs, Sebastian Rachuba, Gréanne Leeftink

**Affiliations:** 1 Center for Healthcare Operations Improvement and Research (CHOIR), University of Twente, Drienerlolaan 5, Enschede, The Netherlands; 2 Department of Internal Medicine, Isala, Zwolle, The Netherlands; 3 Connected Care Center, Isala, Zwolle, The Netherlands; 4 Medical School, University of Exeter, St Luke’s Campus, Exeter, UK

**Keywords:** Outpatient clinics, Planning & scheduling, Appointments, Patient flow, Avoidable assessments, Pre-assessment

## Abstract

**Background:**

Outpatient Clinics (OCs) are under pressure because of increasing patient volumes and provider shortages. At the same time, many patients with chronic diseases receive routine follow-up consultations that are not always necessary. These patients block access to care for patients that are in actual need for care. Pre-assessing patient charts has shown to reduce unnecessary outpatient visits. However, the resulting late cancellations due to the pre-assessment, challenge efficient alignment of capacity with actual patient demand, leading to either empty slots or overtime.

This study aims to develop a method to analyse the effect of pre-assessing patients before inviting them to the OC. This involves 1) to select who should come and 2) to optimize the impact of pre-assessment on the schedule and efficient use of OC staff.

**Methods:**

This prospective mixed-methods evaluation study consists of 1) an expert meeting to determine a pre-assessment strategy; 2) a retrospective cohort study to review the impact of this strategy (12 months of a Dutch nephrology OC); 3) mathematical optimization to develop an optimal criteria-based scheduling strategy; and 4) a computer simulation to evaluate the developed strategy. Primary outcomes are the staff idle time and staff overtime. Secondary outcomes evaluate the number of weekly offered appointments.

**Results:**

The expert group reached consensus about the pre-assessment criteria. 875 (18%) of the realized appointments in 2022 did not meet the OC visit pre-assessment criteria. In the best performing scheduling strategy, 94 slots (87% of the available capacity) should be scheduled on a weekly basis. For this schedule, 26.8% of the OC weeks will experience idle time ($\mu$=2.51, $\sigma$=1.44 appointment slots), and 21% of the OC weeks will experience overtime ($\mu$=2.26, $\sigma$=1.65 appointment slots) due to the variation in patient appointment requests. Using the pre-assessment strategy combined with the best performing scheduling strategy under full capacity (108 slots), up to 20% increase in patient demand can be handled with equal operational performance.

**Conclusions:**

This evaluation study allows OC managers to virtually test operational impact of pre-assessment strategies on the capacity of their OC, and shows the potential of increasing efficient use of scarce healthcare capacity.

**Trial registration:**

Not applicable.

**Supplementary Information:**

The online version contains supplementary material available at 10.1186/s12913-024-11615-7.

## Background

Outpatient Clinics (OCs) are under pressure because of increasing volumes and capacity constraints. At the same time, many patients with (chronic) disease get routine OC follow-up consultations that are not always necessary [1, 2]. Research showed that actively pre-assessing patient charts before scheduled outpatient visits, and cancelling unnecessary outpatient visits, can increase outpatient department effectiveness and can reduce unnecessary outpatient department attendances by up to 40% [1]. These are patients that do not necessarily need to be discharged from the OC (end of care), but at this point in time do not need additional attention as for example there would be no change in management or information provision, or no change in disease status present [3].

As a result, these patients prevent access to care for other patients, such as new patients or patients with significant disease progress, and cause an unnecessary increase in workload of healthcare practitioners. It is long-time recognized that a reduction in the numbers of follow-up patients might improve the service offered to e.g., newly referred patients [4, 5], and that avoiding unnecessary referrals is important given the limited capacity in healthcare providers [6–8]. Therefore, it is essential to analyze outpatient follow-up appointments, and establish a framework that allows their reduction without impacting patient care quality [4].

When identifying which patients scheduled for follow-up care are in actual need of an appointment, a change in operational OC performance (e.g., utilization of the clinic) is expected. In general improved operational effectiveness is expected through more time for relevant patients. However, at the same time, pre-assessment based invitations also increase the variability in patient arrivals, resulting in higher expected overtime and idle time [9]. Patients enrolled in traditional care pathways typically know well in advance when they will obtain a follow-up appointment (e.g., every 3 months or every year) independent of their health status at that point in time. Temporary mismatches in supply (number of available slots) and demand (patient appointment requests) can therefore easily be diverted by earlier inviting or delaying some patients, or by adapting capacities. In a criteria-based planning strategy it is only known on a short-term basis whether that patient should be invited depending on certain criteria, resulting in *advanced access* or *open access* policies where patients receive an appointment quickly following the moment of presentation, for example on the day in which they present themselves [10]. This criteria-based planning on short notice challenges efficient planning. The scheduling flexibility is more limited, since capacities cannot be easily changed anymore, and the actual amount of patients in need of a consultation is only known on short notice. This might cause increased overtime on some days, while other days increased idle time will be observed [11]. On the other hand, the reduction in patient volume might offer extra flexibility to schedule those patients.

In this study, we propose a mixed-methods approach to analyze the potential effects of pre-assessing patient statuses before inviting them to the OC on the operational efficiency of an OC. In this approach we 1) identify a pre-assessment strategy, and 2) assess the expected impact of criteria-based advanced scheduling on the schedule. We exemplify this approach in a case study of the nephrology OC in a Dutch hospital.

Pre-assessment of patient charts aims to reduce unnecessary outpatient visits. Historically, patient charts were hand-screened, but nowadays automated screening of patient charts for irregularities, or remote monitoring of patients, offer opportunities to increase on-demand care provision, and to reduce unnecessary outpatient visits due to standardized care-pathways. An example is the use of an Early Warning Score system to monitor vital signs in inpatient wards [12, 13]. When the heart rate or blood pressure exceeds a certain threshold, an indication is given to the caregivers to check on that patient. Note that these data-driven models are often site-specific, due to the inherent dependency of data and contextual criteria representing the local population [12–14]. In nephrology OCs, patients with severe or progressing Chronic Kidney Disease (CKD) receive (long-term) care from a nephrologist. These patients are typically enrolled in a standardized care pathway, with regular follow-up consultations. As the prevalence and incidence of CKD are rising, so are the number of CKD patients in secondary OC care [8, 15]. Although national guidelines exist for referring a CKD patient from primary care to secondary care [16] and recently criteria were developed for referral from secondary care back to primary care [8], no criteria are known for when patients need active consultation with a nephrologist in the long-term secondary care system. This study will therefore develop these criteria.

In order to assess the expected operational impact of scheduling the OC based on these developed criteria in practice, an intervention in the planning strategy is required, to ensure the increased uncertainty in patient demand and reduced flexibility in capacity alignment options have minimal effect on the OC efficiency. For the optimization of planning strategies in healthcare, Operations Research techniques, such as Stochastic Programming, are used to optimize and prospectively analyze the impact of blueprint schedules on various outcome measures, including overtime and idle time, before actual experimentation and implementation in practice [17, 18]. In blueprint design, which is particularly relevant for advanced access systems, resources are allocated to various types of patients, with the objectives of e.g., reducing delays in OC care by protecting time for new appointments [19], reserving appointment slots for walk-in patients [20] or considering unpunctuality of patients [21]. In order to deal with varying demand arrivals, stochastic programming based blueprint designs propose a blueprint that is optimal under various possible patient demand scenarios [22]. Since mathematical models cannot include all details, literature combines these with prospective computer simulation models to give very accurate insight in system behavior on managerial and operational levels. Examples of this include the optimization of releasing reserved capacity to deal with no-shows [23], and considering seasonality while reserving slots for walk-in patients [24]. To the best of our knowledge, despite the large effect on operational efficiency, the impact of appointment pre-assessment on OC efficiency has not been addressed in the literature.

This study aims to design and evaluate the impact of an intervention based on consensus-based pre-assessment in combination with mathematical modeling and computer simulation, to optimize and evaluate OC’s operational efficiency for a Dutch nephrology department. Through this case, we introduce a novel mixed-methods approach to explore the impact of criteria-based planning on the effective use of OC capacity.

## Methods

### Design

This study is a prospective mixed-methods evaluation study of a pre-assessment and criteria-based scheduling intervention. First, the intervention is developed using consensus-based pre-assessment and Stochastic Programming techniques. Second, the intervention is evaluated using computer simulation, to assess likely benefits while not inferring with clinical practice.

Ethics approval was provided on 12-05-2023 by the Isala Institutional Review Board board, registration number 20230503, and on 14-05-2023 by the University of Twente-BMS Domain Humanities and Social Sciences Institutional Review Board, registration number 230803. As this project was initiated in the quality and improvement scheme, the Institutional Review Board of Isala (registration number 20230503) waived the need for written informed consent from the involved expert team. De-identified patient data was only included if patients gave general informed consent at hospital level for the use of their data for research & education.

### Case study setting

The intervention was implemented in a nephrology clinic of an Internal Medicine (IM) department of a large Dutch regional hospital. In this clinic, patients with CKD are treated in an outpatient setting. CKD is defined as the presence of an abnormal kidney function (estimated Glomerular Filtration Rate (eGFR)) and/or a marker of kidney disease (e.g., Albumin-Creatinine Ratio (ACR)), for at least three months [25]. CKD stages are defined by both eGFR (G1-5) and albuminuria (A1-3). Staging CKD by eGFR and albuminuria not only indicate the severity of kidney disease, but are also related to the risk for End Stage Renal Disease (ESRD) and cardiovascular morbidity and mortality.

During the study period, the clinic was run by 6 nephrologists, with varying availabilities depending on secondary tasks. All nephrologists of the department were included in the stakeholder consultation.

OC consultation hours are built up out of blocks of three hours. The current weekly blueprints for these blocks consist of multiple types of appointments, of which we include three in this analysis:

- New patient appointment (duration 30 minutes)
- Follow-up patient appointment (duration 15 minutes)
- Telephonic consultation (duration 15 minutes)

For follow-up patient appointments, 108 time slots of 15 minutes are available on a weekly basis (ranging from 0-26 per nephrologist). These appointments are typically scheduled 3-6 months in advance, following a standardized care pathway protocol.

Data were retrospectively collected over 12 months, from January 1st to December 31st 2022. This data consisted of all nephrology patients that had a recurring visit to the OC in 2022, the number and type(s) of their appointment(s), together with the patient’s blood pressure, eGFR, ACR/Protein-Creatinine Ratio (PCR), sodium, potassium, bicarbonate, calcium, phospate, Parathormone (PTH), hemoglobin and ferritin for each individual appointment. Note that patients who were treated in primary care were excluded in this analysis. Patients who did not consent to their data made available for research use were not included in the dataset (<0.1% of total sample).

### Intervention

We developed an intervention that consists of two phases: 1) the pre-assessment strategy and 2) a corresponding criteria-based scheduling strategy.

#### Pre-assessment strategy

An OC pre-assessment strategy was designed based on the Dutch CKD guidelines for primary care [16] and discussed in an expert meeting to derive general consensus of patient criteria for an outpatient visit. In this expert meeting with nephrologists (*n*=6) of Isala Hospital the outpatient visit pre-assessment criteria were defined. Afterwards, a retrospective cohort study was performed reviewing records of all patients under nephrologist outpatient care in 2022.

Descriptive statistics were used to assess the patients’ characteristics and the proportion of patients visiting the nephrology clinic who were not in need of an OC visit. Independent z-tests were completed for subgroup comparisons, and statistical significance was considered for $p\le$0.05.

#### Criteria-based scheduling strategy

The optimal scheduling strategy given the pre-assessment-based patient invitation to the OC was thereupon determined using a Stochastic Programming model with scenario sampling. Stochastic programming is an optimization approach that can identify optimal scheduling strategies while taking into account the effects of these strategies on potential patient demand scenarios. We developed a Stochastic Programming model which determines an optimal blueprint schedule.

The *objective* of the model is to find the blueprint that minimizes the expected number of empty slots, the expected chance of working in overtime, and if so, the amount of overtime. Note that our model can be adjusted by using various weight settings; each OC can individually determine the importance of these various objectives. For our setting, minimizing overtime was considered 2 times more important than minimizing idle time.

The *input* parameters for our model are the distributions of weekly patient demand for each patient type, the available capacity per week for each staff member, the objective weights, and the allowed probability of running in overtime. The input data for the intervention is presented in Appendix A.

We formulated the following *constraints* as restrictions to the final blueprint:

- Regular slots can only be planned within the available capacity;
- Empty slots are regular slots that were planned in the blueprint, but are not used by any patient;
- Overtime slots are those slots that are needed to accommodate all patients, but exceed the regular slots (including those outside available capacity);
- Overtime can only occur with probability $\alpha$, where $\alpha =0.1$ for our setting based on stakeholder consensus.

The *output* of the model is an OC blueprint at patient type level and provides the number of regular slots per type to be planned on a weekly basis. Furthermore, for each potential realization of patient demand, it shows how that particular week would have looked like with respect to empty slots and overtime.

The Stochastic Programming model formulation is presented in Appendix B.

### Prospective evaluation

The quantitative evaluation of expected performance in OC practice of the proposed intervention was derived via Monte-Carlo (MC) simulation, a frequently used computer simulation method in healthcare [26].

In the MC simulation, the blueprints of the SP model are implemented for all simulated weeks. Each week in the simulation, patients arrive according to the empirical distribution as derived from historical data (see Appendix A). On patient arrival, if possible, the patient is scheduled in the first available regular slot of the blueprint of the corresponding week. If no such slot is available, the patient is assigned to a slot in overtime for that week.

The simulation was run for 10,000 independent iterations of one week, and analyzed by descriptive statistics. The following settings were compared:

1. **current population**: intervention without pre-assessment of OC visits but with advanced scheduling in optimal blueprint;
2. **pre-assessed population**: intervention with pre-assessment of OC visits and advanced scheduling in optimal blueprint;
3. **pre-assessed population with increased demand**: intervention with pre-assessment of OC visits and advanced scheduling in optimal blueprint, given a demand increase of 10%, 20%, 30%, and 40%.

### Outcome measures

To measure the expected operational efficiency of the OC, we defined the primary outcomes in the prospective evaluation as the expected staff idle time and staff overtime. The idle time and overtime were measured in appointment slots per week (reported by average and standard deviation), and represent the difference between the actual number of patient appointments requested in a week and the number of appointment slots planned in the blueprint. Idle time is incurred if more slots are planned than patients arrive in a certain week, overtime is incurred if fewer slots are planned than patients arrive in a certain week. The idle time and overtime were furthermore measured in the expected fraction of weeks in which idle time and overtime is incurred.

Secondary outcomes evaluated the number of weekly planned appointments in the blueprint, measured by the number of slots per week. Note that only regular slots are included, so no overtime slots are accounted for.

## Results

This section presents the results of the pre-assessment strategy, the scheduling strategy, and the intervention outcomes under potential demand increase scenarios.

### Pre-assessment strategy

The expert group reached consensus about the pre-assessment criteria, which were based on the Dutch CKD guidelines for primary care [16]. For patients with a CKD diagnosis, the pre-assessment criteria were based on the definition of their diagnosis [25]. Patients with CKD with an eGFR $\ge$45 ml/min/1,73 m2 (G1-3a) and ACR $\le$30 mg/mmol (A1 and A2) or an eGFR 30-45 ml/min/1,73 m2 (G3b) and ACR $\le$3 mg/mmol (A1) should be referred back to primary care and therefore do not require an OC nephrology visit. For the CKD patients that did not meet the aforementioned criteria, and all remaining nephrology patients without a CKD diagnosis, the criteria of Table 1 were used to determine the need for an OC visit. If a measurement was not present, it was assumed that there was no indication for such measurement, and therefore, no visit is required based on that criterion.

**Table 1:** Pre-assessment criteria for nephrology OC

Criterion	No OC visit required	Manual inspection required	OC visit required
Progression eGFR (ml/min/1,73m2)	<15%^a^	-	$\ge$15%^a^
ACR (mg/mmol)	<3.0	3-30	$\ge$30
PCR (mg/mmol)	<0.15	0.15-0.5	$\ge$0.5
Syst. Blood pressure (mmHg)	101-130	131-140	$\le$100 or $\ge$141
Potassium (mmol/l)	3.5-5.5	3.0-3.4	<3.0 or >5.5
Calcium (mmol/l)	2.20-2.65	2.10-2.19	<2.10 or >2.65
Phosphate (mmol/L )	<1.50	1.50-1.80	>1.80
PTH (mg/mmol)	7.0-35.0	<7.0 or >35.0	-
Bicarbonate (mmol/L)	$\ge$20.0	18.0-19.9	<18.0
Natrium (mmol/L)	135-145	130-134	<130 or >145
Hemoglobine(mmol/L)			
*No use of Darbepoetin*	M: $\ge$8.5, F: $\ge$7.5^b^	M: <8.5, F: < 7.5	-
*Use of Darbepoetin*	6.2-7.4	-	<6.2 or > 7.4
Ferritin ($\mu$g/l)	$\ge$ 100	-	< 100

^a^Compared to previous measurement outcomes. ^b^Gender specific

Table 2 shows the retrospective analysis over all appointments of 2022. Overall, 4,821 OC appointments of 1,898 individual patients took place in 2022. 875 of the 4,821 appointments (18.17%) met the *no OC visit required* pre-assessment criteria, indicating no medical indication for an OC visit. Another 124 appointments (2.57%) required manual inspection.

**Table 2:** Characteristics of appointments for all patients, and for patients who did and did not meet the pre-assessment criteria in the nephrology OC

	All unique appointments^a^	Appointments meeting criteria^a﻿^	Appointments not meeting criteria^a﻿^	*P*-value
		*Appointment needed*	*No appointment needed*	
	n=4821	n = 876	n=3945	
# Unique patients	1898	522	1593	
Gender, female (%)	36.29%	46.63%	34.00%	<0.001
Age in years	67.68 (±﻿ 13.87)	64.90 (± ﻿14.45)	68.29 (±﻿ 13.66)	<0.001
	n=4821	n=876	n=3945	
eGFR (ml/min/1.73m2)	38.66 (± 21.82)	46.69 (± 22.00)	36.88 (± 21.38)	<0.001
	n=4821	n=876	n=3945	
ACR (mg/mmol)	62.74 (± 133.20)	5.32 (± 6.88)	68.99 (± 138.82)	<0.001
	n=2403	n=236	n=2167	
PCR (mg/mmol)	1.00 (± 1.49)	0.20 (± 0.21)	1.06 (± 1.53)	<0.001
	n=3349	n=228	n=3121	
Syst. blood pressure (mmHg)	132.72 (± 18.81)	132.07 (± 19.65)	132.87 (± 18.62)	0.326
	n=3757	n=688	n=3069	
Potassium (mmol/l)	4.20 (± 1.71)	4.30 (± 3.11)	4.40 (± 1.20)	0.696
	n=4805	n=874	n=3931	
Calcium (mmol/l)	2.37 (± 0.12)	2.38 (± 0.11)	2.37 (± 0.12)	<0.001
	n=4598	n=781	n=3817	
Phosphate (mmol/L)	1.08 (± 0.67)	1.05 (± 0.27)	1.09 (± 0.72)	0.013
	n=4441	n=727	n=3714	
PTH (mg/mmol)	12.09 (± 8.81)	12.39 (± 10.96)	12.04 (± 8.39)	0.484
	n=3651	n=537	n=3114	
Bicarbonate (mmol/L)	24.41 (± 3.14)	24.92 (± 3.19)	24.33 (± 3.13)	<0.001
	n=3412	n=474	n=2938	
Natrium (mmol/L)	139.58 (± 3.01)	139.55 (± 3.17)	139.59 (± 2.97)	0.739
	n=4808	n=874	n=3934	
Hemoglobin (mmol/L)	8.08 (± 1.18)	8.29 (± 1.08)	8.04 (± 1.19)	<0.001
	n=4740	n=852	n=3888	
Ferritin ($\mu$g/l)	227.93 (± 255.28)	199.79 (± 184.08)	232.18 (± 264.17)	0.020
	n=1750	n=230	n=1520	

^a^values displayed as mean (± standard deviation), *n* measurements, unless otherwise stated

The characteristics of the patients of the appointments who did not meet the criteria were: 34.0% female, a median age of 66, 87.8% was diagnosed with CKD, and a mean eGFR of 36.9 ml/min/1,73m2. The most common stage (19.3%) was G4 (eGFR 15-29) and ACR unknown, which means that the there was no indication for a measurement.

### Effects of intervention

Table 3 presents the expected effects on the scheduling efficiency of the OC given the intervention.

**Table 3:** Expected outcomes of optimal blueprints for criteria-based appointment scheduling

	Current population^a^	Pre-assessed population				
*Demand increase*	*0%*	*0%*	*10%*	*20%*	*30%*	*40%*
*Overtime*						
Fraction of OC with OT	28.0%	21.0%	22.8%	28.9%	32.0%	39.1%
Time^b^, mean$\pm \sigma$	2.98 ±2.41	2.26 ±1.65	2.75 ±2.16	3.35 ±2.67	4.38 ±3.36	5.11 ±3.97
*Idle time*						
Fraction of OC with IT	34.9%	26.8%	27.8%	22.1%	21.8%	15.7%
Time^b^, mean$\pm \sigma$	2.91 ±1.87	2.51 ±1.44	2.71 ±1.69	2.39 ±1.57	2.44 ±1.56	1.99 ±1.17
*Number of slots*						
Regular time slots	102	94	101	103	108	108

^a^these outcomes represent the current population scheduled in an advanced access setting (i.e., access in the week of arrival) - which does not represent the current way of in-advance scheduling in the department ^b^overtime and idle time are presented in number of time slots

The prospective evaluation of the intervention shows that for the best performing schedule, 94 15-minute slots are regularly planned on a weekly basis, which is a capacity decrease of 13.0% (3 hours). For this schedule, 26.8% of the OC weeks will experience idle time ($\mu$=2.51, $\sigma$=1.44 time slots), and 21% of the OC weeks will experience overtime ($\mu$=2.26, $\sigma$=1.65 time slots) due to the variation in patient appointment requests.

In the current situation only a negligible amount of idle and overtime is experienced that is caused by fluctuations in patient demand, as the patient appointment requests are known weeks in advance, so the full OC capacities (108 time slots per week) can be adapted to the required number of appointments. However, if the current patient population would be scheduled in a criteria-based way, 102 slots are planned, for which 34.9% of the OC weeks will experience idle time, ($\mu$=2.91, $\sigma$=1.87 time slots), and 28.0% of the OC weeks will experience overtime ($\mu$=2.98, $\sigma$=2.41 time slots) due to the variation in patient appointment requests. This constitutes a decrease in performance of 8.1 % point and 7 % point respectively.

### Increase in patient demand

Given the decrease in utilization of regular capacity (-13.0%), the nephrology OC could potentially grow in patient population. Table 3 presents the expected effects on the scheduling efficiency of the OC given the intervention with various patient demand increase scenarios. This table shows that with a 10% demand increase similar performance is reached within the available OC capacity compared to the current situation. For a 30% increase in demand the capacity limit of 108 time slots is reached, which is also shown by the significant increase in overtime and decrease in idle time when further increasing patient demand to 40%.

## Discussion

This study has highlighted that combining efficiency improvements in demand and supply go hand in hand. On the demand side, a significant amount of follow-up consultations in a nephrology clinic (18-21%) were found to have been unnecessary, which is smaller than similar observations for e.g., follow-up after surgery (>60% [4]), and IM department (>40% [1]), but in line with the efficiency focus of the studied OC setting. Furthermore, only 2.57% of the appointments needed manual inspection to see whether a consultation was required, showing this data-driven approach is a promising labor-saving intervention. On the supply side, the reduced scheduling horizon incurred by pre-assessment of OC appointments necessitates rethinking the existing use of capacity [27]. Our proposed intervention thus combines efficiency improvements in demand (by pre-assessing whether patient appointments are necessary) and supply (by optimizing the blueprint which dictates capacity deployment). As demand and supply inherently interact, it is important to integrally address them in any efficiency improvement initiative.

Table 2 shows that several parameters are significantly different in both patient groups, and therefore might be good predictors of the usefulness of an appointment. On the other hand, parameters such as systolic blood pressure, sodium, potassium, and PTH may not directly predict patient classification. It is important to understand that the pre-assessment criteria were selected not for their predictive value but for their clinical relevance, as guided by the Dutch CKD guidelines for primary care [16]. These criteria are crucial for identifying the need for clinical intervention. When a single patient’s measurements deviate from the reference values, it signals a potential requirement for treatment adjustment, often leading to a consultation during which the patient is informed of the findings. In this consultation, the healthcare provider and patient work together to explore the underlying causes of the deviations and decide whether, and how, the treatment plan should be modified. All parameters are therefore vital for guiding clinical decisions and ensuring appropriate patient management.

The retrospective data of the outpatient clinic under study enforced a moderate variation of patient demand per week, as seen in Appendix A, due to the manually scheduled spread of patient appointments in the historical situation. In less regulated situations, for example with weekly remote monitoring of disease progress, the variation in patient demand for consultations can be even larger, further reducing the efficiency of the outpatient clinic. The proposed intervention should therefore be used by outpatient clinics to assess their target efficiency using the demand distribution derived from their own clinic’s data. Note that the approach allows any type of distribution, including frequently used distributions such as the Poisson or Normal distributions, but also empirical distributions.

The choice of input parameters could have an influence on the results. To assess this effect, we conducted a sensitivity analysis on the choice of the objective weights and the allowed probability of running in overtime $\alpha$. For the latter, the analysis showed that if $\alpha$ increases, the overtime incidence rate slightly increases and stabilizes at 21% with $\alpha$ = 0.3, showcasing that the workload in our case study is relatively balanced with the available workforce hours. On the contrary, the choice of weights of idle and overtime heavily influences the results. If the minimisation of idle time is preferred over minimising overtime (3:1), the reserved number of slots drops down to 69 slots, and the overtime incidence rate increases to 44%. The expert panel confirmed that the 1:2 ratio was still preferred after seeing these results. However, these large variations in outcomes do confirm that it is important to conduct a sensitivity analysis to validate the choice of input parameters with your stakeholders.

In the intervention, the design of the pre-assessment criteria is based on medical consensus. In certain applications, combining qualitative and quantitative methods could be beneficial, as also applied in other areas of quality improvement in healthcare (e.g., [28]). In the intervention, operations research and data-analytics methods could support such consensus discussions, for example by using machine learning approaches. For our case study, Table 2 already suggests which clinical indicators seem to be good predictors.

To ensure adherence to the criteria-based invitation strategy, it is important in implementing this strategy that patients and providers are well educated about the guidelines and motivation regarding OC visits [4]. Furthermore, clear communication with patients needs to be provided on when they are in need and can expect a visit when entering OC care [4, 29]. Otherwise, disparities might arise between patients’ and clinicians’ perceptions of whether an OC visit is needed (as also observed e.g., for OC discharge [29]), or unnecessary visits take place anyway. Clear communication is also required to clinicians and supporting OC staff regarding the expected organisational performance, as through changing to an advanced access scheduling policy, increased idle and overtime is expected, which reduces the utilization of OC capacity.

As a result of this intervention, the nephrology department implemented a real-time dashboard with the pre-assessment criteria as decision support for the OC. With patients that do not meet the criteria for a visit, cancelling the appointment is discussed on an individual basis. In a follow-up project, patients will, prior to their potential OC visit, receive a questionnaire to determine their need for an appointment. Further research is needed to include the patient perspective on criteria-based appointment scheduling in follow-up OC care.

This study is subject to several limitations. First, we assumed all patients needed a consultation in the week of their arrival. In practice some patients can be delayed by one or two weeks, to alleviate the effects of variability in patient arrivals. We hypothesize that the performance of the schedules when incorporating delays will improve with respect to empty slots and overtime, due to the reduction in demand variability. Further research should analyse these effects to quantify its impact.

Second, the generalizability of the case study outcomes depend on site-specific population data and contextual parameters on e.g., capacity availability. However, other organizations can use the proposed approach, including the developed Stochastic Programming and simulation models, and run it with their own data to obtain relevant organization-specific outcomes, significantly reducing the required time investment for such an analysis.

Third, the expert group reached consensus on three categories: patients that did not need an OC visit, patients that did need an OC visit, and patients for which a manual inspection of the patient chart was required. In the scheduling evaluation, we considered this third group as in need of an OC visit. In the expert group meeting, nephrologists indicated that in their experience they expected that for a reasonable portion of this group the OC visit did not add value for their course of treatment and clinical outcomes, but that further tightening of the criteria was hard while ensuring the quality of care was not jeopardized. As this group only represents 2.57% of the total number of appointments, there is still some potential in further reducing unnecessary appointments, but without major expected impact on operational efficiency.

Fourth, it is important to consider the impact of the outpatient visit on the patient as a whole, and not to only rely on medical selection criteria. As mentioned, patient preferences will therefore be included in a pre-assessment questionnaire, to assess the patient’s need for an appointment. Note that while objective criteria are relatively easy to interpret, more subjective criteria can both result to appropriate and inappropriate influences, as also e.g., seen in discharge decisions [30].

Fifth, the pre-assessment criteria in this study are applied in isolation, and not yet adapted to the patient’s individual needs, history, and its possibilities in therapeutic options. As an example, consider a patient with diabetic nephropathy and a neatly regulated blood pressure, who is adequately adjusted on all required medication, but still has a protein loss (ACR) >30 mg/mmol, in line with previous visits. According to the defined criteria, this patient should be scheduled for an OC visit. However, there are no additional therapeutic options, and a consultation does not have any added value to the patient’s care pathway. This shows a potential for further reduction in number of consultations, and need for further research in personalized pre-assessment criteria.

## Conclusions

By introducing automated pre-assessment of patient charts in combination with criteria-based scheduling, serving an 20% increase in patient demand is possible for the nephrology OC of Isala Hospital, through increasing efficient use of scarce healthcare capacity. 18% of OC appointments in the nephrology department in 2022 was considered unnecessary.

With the advanced access planning of the remaining patient population, efficient planning will be increasingly difficult, through the reduced flexibility to match variations in patient demand with provider capacity. For the nephrology department under study our criteria-based scheduling strategy will result in some overtime and idle time in respectively 21% and 25% of the weeks, and is robust for a patient demand increase of over 20%.

Our mixed-methods approach allows OC managers to develop test potential pre-assessment strategies before an actual implementation, without disrupting the ongoing workflow. Furthermore, it informs OC decisions on capacity allocation, to achieve a stable workload and operational efficiency despite the reduced flexibility in matching patient demand with provider capacity.

## Supplementary Information

Supplementary Material 1.

## Data Availability

Data is available with the authors upon reasonable request and with hospital permission.

## Abbreviations

ACR: Albumin-Creatinine Ratio
CKD: Chronic Kidney Disease
ESRD: End Stage Renal Disease
eGFR: estimated Glomerular Filtration Rate
IM: Internal Medicine
MC: Monte-Carlo
OC: Outpatient Clinic
PCR: Protein-Creatinine Ratio
PTH: Parathormone
