# Supplementary material for: Criteria-based outpatient scheduling at a nephrology clinic: prospective evaluation of patient pre-assessment and its corresponding adaptive scheduling strategy
Source: BMC Health Serv Res. 2024 Sep 28;24:1145. doi: 10.1186/s12913-024-11615-7 (PMC11437885; doi:10.1186/s12913-024-11615-7)
Supplement: Supplementary file 1 — Supplementary Material 1. [file 12913_2024_11615_MOESM1_ESM.pdf]

Appendix A Input data for Stochastic Programming model

A.1 The distribution of weekly demand of patients for each patient type

The patient demand follows a normal distribution, truncated ≥ 0, with parameters as displayed in Table A1.

Table A1: Parameters of weekly demand of recurring outpatient clinic Chronic Kidney Disease (CKD) and non-CKD patients per patient type

**Patient type**	μ, σ
CKD-Red	37.50, 10.20
CKD-Yellow	19.60, 6.20
Non-CKD-Red	14.80, 4.58
Non-CKD-Yellow	8.29, 3.90
Other	14.31, 4.59

A.2 The available capacity per week per nephrologist

The average available capacity per nephrologist per week for recurring outpatient consultations is provided in Table A2, as based on historical data of January - December 2022.

Table A2: Available capacity per week per provider

**Nephrologist**	**Capacity (#slots)**
Nephrologist 1	17
Nephrologist 2	8
Nephrologist 3	22
Nephrologist 4	19
Nephrologist 5	16
Nephrologist 6	26

Appendix B Stochastic Programming model for criteria-dependent outpatient clinic planning

Table B3 presents the model's sets, parameters, and variables. Given this notation, the Stochastic Programming model can be found in (B1)-(B11).

Table B3: The sets, parameters, and variables of the flexible nurse and patient allocation optimization model

Index and set	Definition
$i \in I$	set of patient types
$r \in R$	set of resources
$s \in S$	set of scenarios

Parameter	Definition
$d_{r,s,i}$	weekly demand of patient type i at internist r in scenario s
c_r	available appointment slots per week of internist r
α	parameter to set the max percentage of overtime slots
$\gamma_1, \gamma_2, \gamma_3$	weights for (1) overtime, (2) idle time, and (3) feasibility penalties in objective

Variable	Definition	Range
$X_{r,i}$	Number of slots for patient type i at resource r	integer
$Y_{r,s,i}$	Number of slots for patient type i at resource r used in scenario s	integer
$P_{r,s,i}$	Number of empty slots for patient type i at resource r in scenario s	integer
$O_{r,s,i}$	Number of slots in overtime for patient type i at resource r in scenario s	integer
$Z_{r,s,i}$	Indicator variable whether extra slots are needed (1) or not (0) for patient type i at resource r in scenario s	binary
B	Indicator variable whether α is met (1) or not (0)	binary

Objective

$$\min \quad \sum_{r,s,i} \gamma_1 P_{r,s,i} + \gamma_2 O_{r,s,i} + \gamma_3 B\left(\frac{1}{|R||S||I|} \sum_{r,s,i} Z_{r,s,i} - \alpha\right) \tag{B1}$$

Constraints

$$\sum_{i} X_{r,i} \leq c_r \qquad \forall r \in R \tag{B2}$$

$$Y_{r,s,i} \leq X_{r,i} \qquad \forall i \in I, r \in R, s \in S \tag{B3}$$

$$Y_{r,s,i} + O_{r,s,i} = d_{r,s,i} \qquad \forall r \in R, s \in S, i \in I \tag{B4}$$

$$P_{r,s,i} = X_{r,i} + O_{r,s,i} - d_{r,s,i} \qquad \forall r \in R, s \in S, i \in I \tag{B5}$$

$$\frac{1}{|S||I|} \sum_{r,s,i} Z_{r,s,i} - \alpha < MB \tag{B6}$$

$$\alpha - \frac{1}{|S||I|} \sum_{r,s,i} Z_{r,s,i} < M(1 - B) \tag{B7}$$

$$O_{r,s,i} \leq M Z_{r,s,i} \qquad \forall r \in R, s \in S, i \in I \tag{B8}$$

$$Z_{r,s,i} \leq O_{r,s,i} \qquad \forall r \in R, s \in S, i \in I \qquad \text{(B9)}$$

$$Z_{r,s,i}, B \in \{0,1\} \qquad \forall r \in R, s \in S, i \in I \qquad \text{(B10)}$$

$$X_{r,i}, Y_{r,s,i}, O_{r,s,i}, P_{r,s,i} \in \mathbb{Z}^+ \qquad \forall r \in R, s \in S, i \in I \qquad \text{(B11)}$$

The objective (B1) minimizes the expected weighted number of empty slots, number of slots in overtime, and the penalty of running in overtime in more than α percent of the time.

Constraints (B2) only allow the blueprint to schedule available Outpatient Clinic (OC) capacity. Constraints (B3) check for each scenario enough how many regular slots are used. Constraints (B4) ensure that for each demand scenario all patients are scheduled (either in a regular slot (Y) or an overtime slot (O)), and Constraints (B5) ensure that all regular slots are accounted for (either occupied by a patient Y or empty P). Constraints (B6) and (B7) ensure that not more than α percent of the scenarios will run in overtime, where Constraints (B8) and (B9) set the indicator variable whether there is overtime in a scenario or not.

A blueprint is derived based on sampling 1,000 scenarios from the input data distributions in Appendix A. The blueprint representing the rounded average of six runs is chosen for further evaluation.
